# Emissions Reductions to Meet a Tighter Ozone Standard in the U.S. through Control Technologies versus Clean Energy Transition Scenarios

**DOI:** 10.1021/acs.est.5c01588

**Published:** 2025-12-15

**Authors:** Paul Meier, Tracey Holloway, Xinran Wu, Cecilia Orth

**Affiliations:** † Center for Sustainability and the Global Environment (SAGE), Nelson Institute for Environmental Studies, 5228 University of Wisconsin−Madison, 1710 University Avenue, Madison, Wisconsin 53726, United States; ‡ Department of Atmospheric and Oceanic Sciences, University of Wisconsin−Madison, 1225 West Dayton Street, Madison, Wisconsin 53706, United States

**Keywords:** multipollutant, cobenefits, pollution control, climate change, multipollutant control pathways

## Abstract

Past studies have found that carbon reduction strategies generally reduce emissions of nitrogen oxides (NO_X_) and/or volatile organic compounds (VOCs). The reverse is not true, however, as evidenced by over 50 years of air quality improvements in the U.S. with only modest reductions of carbon dioxide (CO_2_) emissions. This analysis compares energy and emissions pathways to achieve NO_X_ and VOCs targets calculated by the U.S. Environmental Protection Agency (EPA) to meet a hypothetical 65 ppb revision to the National Ambient Air Quality Standard (NAAQS) for ozone. To meet these targets, we model sector-specific reductions over a 15-year horizon with the Multipollutant Emissions Calculator for Air Quality and Climate (MECAQC), considering both conventional emission controls, e.g. technologies, as well as energy system changes, e.g., fuel-switching. Switching away from conventional fuels can achieve the majority of required NO_X_ and VOCs emission reductions, considering maximum decarbonization up to 45% for heavy-duty vehicles, up to 65% for light-duty vehicles, up to 36% for building electrification, and up to 100% for electricity generation. The maximum decarbonization assumptions alone could meet NO_X_ and VOCs targets in the Midwest region and VOCs targets in the Northeast region, reducing NO_X_ emissions in affected areas by 44%, VOCs by 16%, and CO_2_ by 56%. These carbon-reduction strategies may be supplemented by conventional emission controls to achieve additional VOCs reductions to meet the most targets: reducing NO_X_ emissions in affected areas by 33%, VOCs by 17%, and CO_2_ by 35%. Conventional controls alone could meet all regional targets except the California NO_X_ target, which cannot be met by any approach evaluated here, reducing NO_*x*_ emissions in affected areas by 33% and VOCs by 17%, but increasing emissions of CO_2_ by 1.4%.

## Graphical abstract

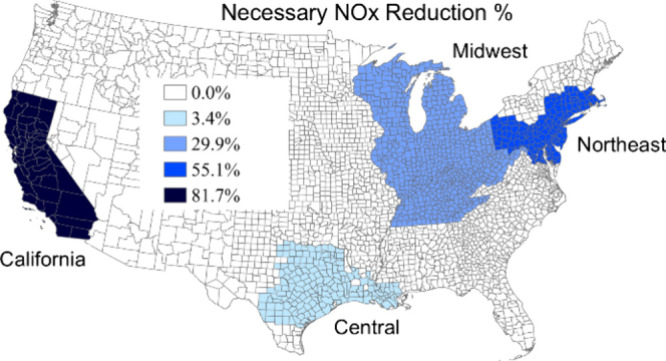

## 1 Introduction

Tropospheric ozone (O_3_) is a secondary pollutant generated by photochemical reactions among nitrogen oxides (NO_X_ = NO + NO_2_) and volatile organic compounds (VOCs), which are emitted from both anthropogenic and natural sources.^1,2^ The U.S. Environmental Protection Agency (EPA) sets National Ambient Air Quality Standards (NAAQS) for ozone and five other criteria pollutants,^3^ primarily to protect public health. Health impacts of ozone exposure include irritation to eyes, nose, and throat; impaired lung function; aggravation of bronchitis, emphysema, and asthma; and other cardiovascular and respiratory impacts, including premature mortality.^4–6^ Studies have also quantified environmental harms,^6–8^ and economic harms,^9,10^ associated with ozone exposure, including the benefits of a stricter ozone standard.^11^ Although ozone regulations have been successful in lowering peak concentrations,^12–14^ adverse impacts on human health persist.^15–19^

States respond to the NAAQS by developing State or Tribal Implementation Plans (SIPs/TIPs), which outline strategies to attain and maintain compliance with the standards. To meet the ozone NAAQS, SIPs must reduce in ozone precursor emissions of NO_X_ and/or VOCs. The specific sources contributing to NO_X_ and VOCs vary by location, with U.S. average NO_X_ emissions over 90% anthropogenic, primarily from fuel combustion such as vehicle transport, industrial sources, and electric utilities.^20^ U.S. average VOC emissions are partially anthropogenic (26%), primarily from vehicle transport and industrial sources,^21^ with biogenic sources, especially trees, contributing the majority of emissions (74%).^22,23^ The effectiveness of NO_X_ versus VOCs emission controls depends on the reactivity of VOCs, the ratio of NO:NO_2_ in NO_X_, and other characteristics of the chemical environment.^2,24,25^ As a result, ozone control may require controlling NO_X_, VOCs, or both.^26^

Reductions in NO_X_ and VOCs emissions have focused on anthropogenic sources where regulatory agencies can require emission control technologies, fuel standards, and/or compliance reporting (e.g., vehicle inspections, continuous emissions monitoring systems). These traditional pollution control measures are implemented to target a single emissions category, either NO_X_ or VOCs. We refer to such an approach as a single-pollutant compliance pathway (SPCP). While conventional approaches focus on SPCPs, alternate strategies hold the potential to affect emissions of multiple pollutants simultaneously.

A broader literature has examined such multipollutant approaches, highlighting both the potential benefits and trade-offs compared with the single-pollutant pathway. Recent studies, for example, have developed cooperative control strategy for NO_X_ and VOCs;^27^ quantified health risks associated with changes in multiple air pollutants using both a single and multipollutant approach;^28^ and assessed interactions among policies targeting ozone, particulate matter, and greenhouse gases.^29,30^ This literature suggests that integrated strategies such as multipollutant frameworks may achieve greater overall efficiency and public health benefits, motivating the need to compare SPCPs with multipollutant pathways.

Integrated Assessment Models (IAMs) have been widely used in examining such multipollutant control interactions, particularly in climate policy studies which links economics, energy use, and emissions of both greenhouse gases (GHGs) and air pollutants.^31^ IAMs with regional or sectoral detail account for existing air-pollution control policies, such as the Greenhouse Gas and Air Pollution Interactions and Synergies (GAINS) and Global Change Analysis Model for the United States (GCAM-USA).^32,33^ Recent work has advanced the representation of air pollution control policies within IAMs. For example, Shi et al. and Ou et al. extended GCAM-USA with state-level detail to simulate how incorporating existing pollution controls in baseline assumptions substantially affect projected emissions and the cobenefits of climate policies.^34,35^ Other work has demonstrated from a more economic-focused perspective that existing nonclimate pollution controls can influence both the air-quality cobenefits and associated costs and cost-effectiveness of climate policies.^36,37^

This study presents and applies a multipollutant analysis approach to a tighter ozone NAAQS, extending the impact of past EPA analyses to include climate mitigation. To characterize both SPCPs and multipollutant compliance pathways (MPCPs), we present and apply the Multipollutant Emissions Calculator for Air Quality and Climate (MECAQC), which was first applied to SO_2_ by Wu et al.^38^ Here we apply MECAQC to a multisectoral evaluation focused on achieving NO_X_ and VOCs reductions in specific U.S. counties.

While nearly every carbon reduction strategy benefits near-surface ozone,^39^ the opposite is not true, as evidenced by the past over 50 years of air quality trends in the U.S. From 1990 to 2022 emissions of NO_X_ decreased 71%,^40^ whereas CO_2_ only decreased 2% over this time.^41^ Studies have consistently found that carbon policies can lead to air quality cobenefits such as reductions in ground-level ozone.^42–48^ Only a few studies have evaluated how air pollution control policies could impact carbon emissions, including in South Africa,^49^ India,^50^ and China.^51–54^

In 2015, the ozone NAAQS was lowered from 75 to 70 ppb, implemented as the three-year average of the fourth-highest maximum daily 8 h concentration.^55^ The impacts of this revision were evaluated by the EPA’s *Regulatory Impact Analysis of the Final Revisions to the National Ambient Air Quality Standards for Ground-Level Ozone*,^56^ which also evaluated an alternative ozone standard level of 65 ppb. This *Regulatory Impact Analysis* (RIA), modeled 2011 sector- and region-specific emission reductions needed to achieve both the revised ozone standard of 70 ppb and an alternative ozone standard level of 65 ppb, then projected forward to 2025 assuming all areas would reach attainment by 2025, except California (due to the severity of air quality challenges in California).

We quantify how implementation of sector-specific decarbonization strategies could achieve both a hypothetical 65 ppb ozone NAAQS and concurrent reductions of carbon dioxide (CO_2_), a greenhouse gas of concern for global climate change.^57^ We updated the baseline emissions inventory to reflect current conditions, then evaluated options to achieve RIA-calculated emission targets. The MECAQC framework, previously applied to evaluate SO_2_ emission impacts of coal-fired power plants,^38^ is extended here to include NO_X_ and VOC reduction strategies. Scenarios are constructed to compare conventional strategies that reduce NO_X_ and VOCs using targeted pollutant-specific controls against decarbonization strategies that simultaneously reduce NO_X_, VOC, and CO_2_ emissions by eliminating fuel combustion.

Results highlight opportunities to achieve multiple environmental objectives in the implementation of health-based air quality goals, and support decision-making on the design and implementation of future ozone control activities.

## 2 Materials and Methods

### 2.1 Pollution Reduction Pathways

Pollution reduction pathways are developed using the Multipollutant Emissions Calculator for Air Quality and Climate (MECAQC) framework to compare conventional compliance approaches with combined strategies to achieve both carbon reductions and meet a hypothetical 65 ppb ozone standard. MECAQC is an emission evaluation framework that catalogs conventional and alternative pollution control options, such as energy transition technologies for multiple pollutants, including SO_2_, NO_X_, VOCs, and CO_2_. A detailed description of the MECAQC framework, including structure and data source is provided in the Supporting Information (Text S2).

MECAQC includes traditional pollution controls, efficiency losses from control operation, and energy system changes like fuel switching and electrification to extend the capabilities of EPA’s Air Pollution Control Cost Manual and Control Strategy Tool (CoST) to consider a full scope of energy transition pathways and multipollutant impacts. While previous applications of MECAQC focused primarily on SO_2_ reduction in coal-fired power plants in Wu et al.,^58,59^ this study is the first application of MECAQC to evaluate NO_X_, VOCs and CO_2_ emission changes resulting from ozone regulatory impacts.

### 2.2 Updated Baseline

The U.S. EPA’s 2015 RIA is based on a 2025 projected inventory that was developed by adjusting the 2011 National Emissions Inventory (NEI) to reflect anticipated changes to future emissions. Our analysis builds on EPA’s analysis in the 2015 RIA because it provides a transparent, rigorous, policy-relevant estimate of required emission reductions to meet a hypothetical, national 65 ppb ozone standard. Our extension of the EPA RIA analysis should not be taken as precise estimates of particular air quality management scenarios. Rather, we evaluate the hypothesis that moving away from combustion to meet more stringent ozone standards can also drive meaningful GHG reductions.

To reflect changes in emissions and energy systems since the RIA was conducted in 2015, we updated the emissions baseline using the most current data available as of 2021, when this study was initiated. The 2017 NEI provided a foundation for NO_X_ and VOCs, though it does not comprehensively cover CO_2_.^60^ For the electricity sector, we replaced NEI values with 2021 CO_2_ and NO_X_ data from EPA’s Clean Air Markets Program Data (CAMPD).^61^ For other fuel combustion, we substituted 2020 CO_2_ data from the Energy Information Association (EIA). For remaining sectors except for on-road vehicles, we used 2020 GHG estimates from EPA’s GHG Inventory.^62,63^ For on-road vehicles, we used projected 2023 emissions from EPA’s MOVES model.^64^ To make these disparate years and sources consistent, we scaled MOVES and EIA estimates such that total sectoral emissions matched EPA’s official 2021 GHG Inventory. In cases where CO_2_ data were missing, we allocated emissions in proportion to reported NO_X_ or VOCs. This approach creates a single, internally consistent 2021 baseline across pollutants and sectors, as summarized in Supporting Information Table 1.

For sources considered, the process of updating the baseline reflected more current emission levels: reducing total NOx by 23% (from 9.4 million tons to 7.2 million tons) and reducing total VOCs by 8% (from 10.5 million tons to 9.7 million tons). The use of 2020 data for some sectors warrants consideration of pandemic-related emissions changes. Relative to 2023 levels, CO_2_ emissions in 2020 were 1.8% higher for residential buildings, 6.6% lower for commercial buildings, and 0.8% lower for the industrial sector.^65^ Of these discrepancies, the most significant to the study is that emissions reduction potential is underestimated for commercial buildings by roughly 7%. Subsequent updates to this work will substitute with more current sources that are not pandemic affected. We also note that we do not differentiate between the contribution of NO and NO_2_ to NO_X_. From U.S. EPA’s Nitrogen Dioxide/Nitrogen Oxide In-Stack Ratio (ISR) Database, the ratio of NO_2_ to NO_X_ is highly variable by source, but NO is generally the larger contributor.^66^ For 2020 reported data, 75% of sources report an NO_2_/NO_X_ ratio less than 0.21.

To implement this analysis, we calculate the NO_*x*_ and VOCs reductions needed for compliance with the 65 ppb ozone standard. To determine the appropriate reductions, we reconstruct the 65-ppb compliant emission target levels (ETLs) achieved in EPA’s 2015 RIA analysis and compare those levels to the Updated Baseline discussed above. The necessary reductions are calculated for each pollutant and region, by subtracting the ETL (65 ppb compliant levels) from the Updated Baseline. Thus, relative to the Updated Baseline, reductions associated with each scenario would achieve the same regional emission levels as the RIA modeling determined would meet the 65 ppb ozone standard.

### 2.3 Scenarios

As illustrated in Figure 1, four regions require emission reductions to meet a 65 ppb ozone standard, relative to the Updated Baseline: California, Central Midwest, and Northeast. Compliance pathways for each region are examined across five scenarios: (1) Conventional Lowest Cost (CLC) scenario; (2) Conventional Maximum Efficiency (CME) scenario; (3) Total Decarbonization Benchmark (TDB); (4) Faster Partial Decarbonization (FPD); and (5) Slower Partial Decarbonization (SPD).

**Figure 1:**
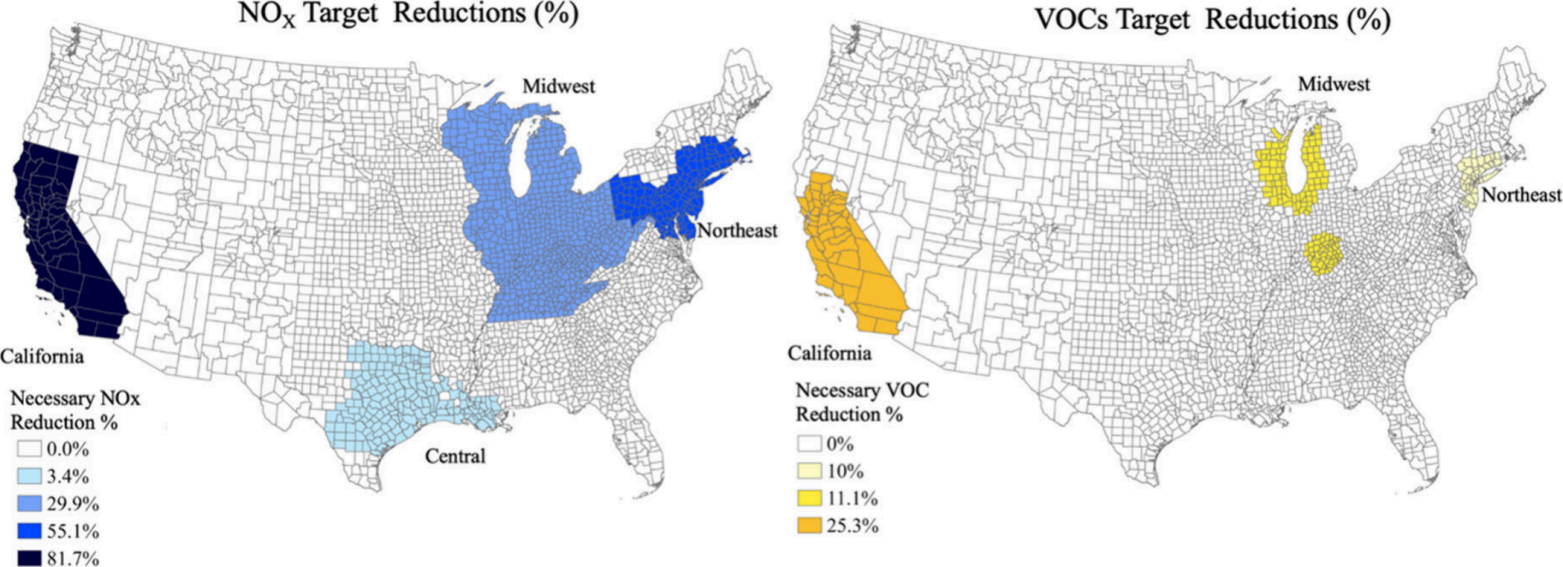
Necessary NO_*x*_ reduction (left, blue) and VOCs reduction (right, yellow) needed to achieve the Alternative 65 ppb Standard Target level (2015 Ozone NAAQS) after updating baseline emissions.

The two conventional scenarios, CLC and CME, apply varying deployments of NO_*x*_ and VOCs pollution controls at the facility level across the four areas where reductions are required. Controls are applied in ranked order starting from the largest emitting source, followed by the second largest emitting source, and so on. The CLC scenario considers each emission source and, of the available control technologies, selects the option with the lowest reported removal cost ($/ton). Controls are applied until the target is met, or in cases where regional targets are not satisfied, until controls are implemented across all affected emission sources. Because the lowest cost technologies frequently have lower control efficiencies, the CLC approach failed to meet emission targets in two regions. The CME scenario considers each emission source and, of the available control technologies, selects the control option identified as having the highest reported control efficiency. Both CLC and CME prioritize control of large sources, and our use of “lowest cost” refers to technology selection for these large sources. It should not be confused with approaches that would economically optimize the lowest cost solution across all sources, although such an approach is of future interest.

The three decarbonization scenarios, TDB, FPD, and SPD, eliminate CO_2_ along with NO_X_ and VOCs emissions at sources where fuel combustion is substituted with nonemitting energy resources. The decarbonization technology substitutions imply that electricity sector reductions derive from renewable or non-emitting electricity, light- and heavy-duty on-road reductions derive from electric vehicles (EVs), and residential and commercial building (furnace/boiler) reductions derive from renewable or non-emitting electric heat. The TDB scenario considers the removal of all fuel combustion emissions from electricity, vehicles and building heating within targeted regions. Because total decarbonization is not likely over the next 15 years, two partial decarbonization scenarios are constructed, FPD and SPD, limiting the maximum rate of decarbonization for each affected sector. For the faster FPD scenario, sectors are limited as follows: 100% decarbonization for electricity (no limit), 45% for heavy-duty vehicles, 65% for light-duty vehicles, and 36% building electrification. For the slower SPD scenario, decarbonization is limited to half the fast sectoral rate: 50% decarbonization for electricity, 22.5% for heavy-duty vehicles, 32.5% for light-duty vehicles, and 18% building electrification.

Both partial decarbonization strategies use decarbonization along with the minimum use of conventional controls required across all regions and standards. Fuel-switching is used preferentially. If necessary, conventional controls are then applied up to the point where regional reduction targets are achieved, or all affected sources are controlled (exhaustion of all sources only occurs in the case of California NO_X_). Conventional controls for these scenarios are selected based on the maximum reported control efficiency, consistent with minimizing the total number of sources requiring emission control additions.

## 3 Results

Based on the EPA’s photochemical modeling to achieve a 65 ppb ozone level presented in the RIA, Emission Target Levels (ETLs) were subtracted from the Updated Baseline emissions in the five study regions (California, Southwest, Central, Midwest, and Northeast) to calculate further reductions needed to meet the hypothetical 65 ppb ozone standard. We refer to this remainder as the Necessary Reduction for each emitted species in each region.

Table 1 shows the Updated Baseline (blue), Emission Target Levels (green), and Necessary Reductions (red). To avoid applying emission controls to many thousands of very small sources, we only consider Necessary Reductions occurring from the top 95% of U.S. NO_X_ and VOCs sources and located in the RIA designated counties (Figure 1), which we refer to as Affected Emissions. Affected Emissions occur in 1,121 counties (out of 3,140 counties in the U.S.) and are responsible for a significant proportion of national emissions: 44% of NO_X_, 13% of VOCs, and 46% of CO_2_. Figure 1 shows the specific counties where Necessary Reductions are calculated, based on figures provided within U.S. EPA’s RIA report (See U.S. EPA RIA’s reported Figures 3–6, 3–7, and 3–10 at the citation provided), as with updated values shown in Table 1.^56^

**Table 1:**
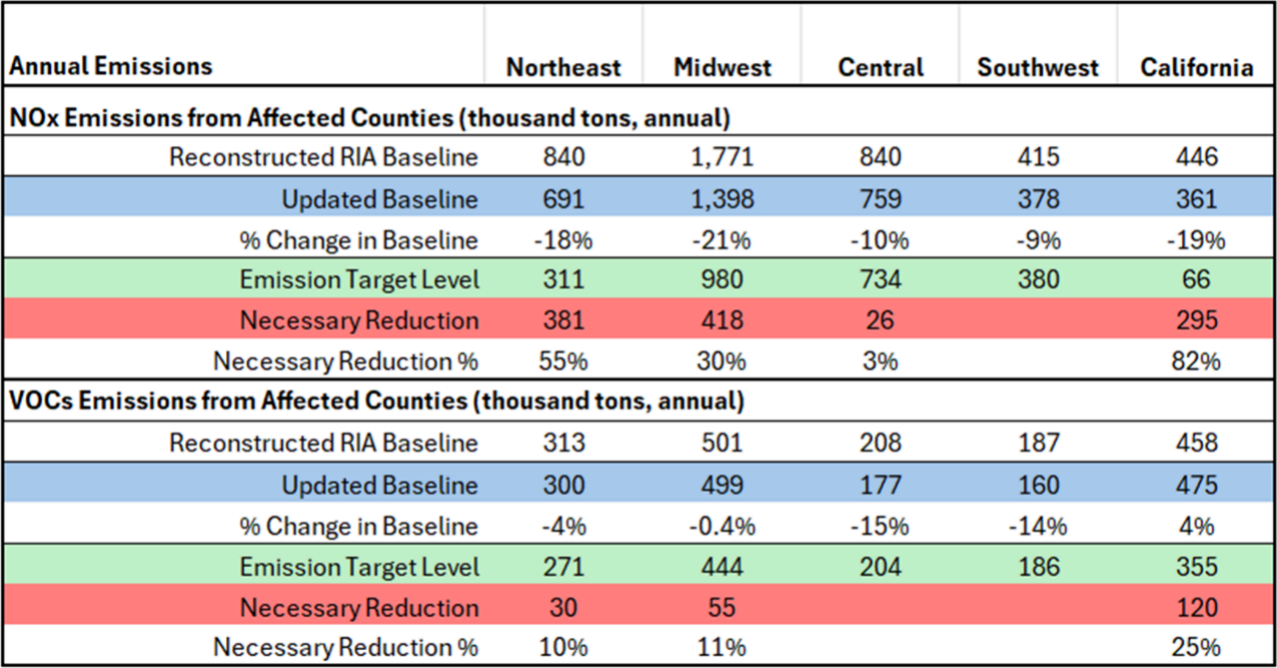
Necessary NO_x_ and VOCs Reductions Needed to Meet the 65 ppb Alternative Standard of EPA’s 2015 Ozone NAAQS Regulatory Impact Analysis (RIA)^a^

a “*Updated Baseline*” denotes the updated emission baseline merged from multiple data sources; “*Emission Target Level*” denotes the levels of the alternative 65 ppb standard derived from the 2015 Ozone NAAQS RIA; “*Necessary Reduction*” and “*Necessary Reduction %*” denotes emission changes in thousand tons and in percentage, required for affected counties to achieve the NO_*x*_ and VOCs Target Levels, respectively.

The RIA uses photochemical modeling of ozone to account for these complex chemical processes, as well as state-to-state transport, and the impacts of emissions outside of the US, which are expected to change since 2011. With high concentrations of vehicles and industrial activity in urban regions, cities tend to have relatively high emissions of both NO_X_ and VOCs, and often the chemical production of ozone in urban areas is VOC-limited.^2,24,25^ In rural areas, NO_*x*_ levels are typically lower so the chemical production of ozone in rural areas is typically NO_X_-limited.^2^ Emission changes for NO_X_, VOCs, and CO_2_ are summarized in Table 2 relative to the Updated Baseline. The CME scenario reduces NO_X_ emissions by 32.9%, VOCs by 16.9%, but increases CO_2_ emissions by 1.4% due to thermal efficiency loss; CLC reduces NO_X_ emissions by 29.9%, VOCs by 16.8%, but also increases CO_2_ emissions by 1.3%. The TDB scenario achieves the highest NO_X_ reduction (43.9%) and CO_2_ reduction (56.4%), while reducing VOCs by 15.6%. The SPD scenario reduces NO_X_ emissions by 33.2%, VOCs by 16.9%, CO_2_ by 21.4%. The FPD scenario reduces NO_X_ emissions by 33.5%, VOCs by 17.2%, and CO_2_ by 34.9%. A regional breakdown of emission changes is provided in Tables 3 and 4.

**Table 2:** Summary of Each Scenario’s Change in Affected Emissions Relative to the Updated Baseline for All Regions^a^

		NO_x_		VOCs		CO_2_	
total emissions change by scenario		ktons	%	ktons	%	Mtons	%
conventional maximum efficiency (CME) using highest efficiency conventional control	CME	–1019	–32.9%	–212	–16.9%	43	1.4%
conventional lowest cost (CLC) using lowest cost conventional control	CLC	–925	–29.9%	–209	–16.8%	39	1.3%
total decarbonization benchmark scenario (TDB) eliminating sector-specific combustion emissions	TDB	–1360	–43.9%	–195	–15.6%	–1672	–56.4%
slower partial decarbonization benchmark (SPD) using sector-specific decarbonization limits	SPD	–1029	–33.2%	–212	–16.9%	–633	–21.4%
faster partial decarbonization benchmark (FPD) using sector-specific decarbonization limits	FPD	–1039	–33.5%	–215	–17.2%	–1035	–34.9%

a Reductions are from electricity, vehicles, and building heat within emission-targeted regions. Each scenario’s achieved percent reduction is relative to affected emissions (sources in the top 95% within affected counties) from the Updated Baseline. Partial decarbonization reflects high and low sensitivity for sector-specific decarbonization limits.

**Table 3:** Regional Breakdown of Annual Emissions from Affected Counties (Affected Emissions), Calculated Emission Target Levels (ETLs), and Regional Emission Levels Achieved in Each Scenario^a^

region	affected emissions	emission target level	CME	CLC	TDB	SPD	FPD
NOx (ktons)							
Northeast	670	289	289	362	336	289	289
Midwest	1328	910	910	910	696	909	906
Central	744	719	718	718	493	717	717
California	356	61	162	184	214	154	147
VOC (ktons)							
Northeast	297	267	265	265	249	264	264
Midwest	484	428	425	427	413	427	424
California	469	349	348	348	393	347	347
CO_2_ (Mtons)							
Northeast	646	0	664	660	233	480	369
Midwest	1239	0	1255	1254	497	896	634
Central	643	0	643	644	303	597	598
California	435	0	444	444	259	356	326

a ETLs refer to the implied levels needed to meet the 65 ppb alternative standard of EPA’s 2015 Ozone NAAQS Regulatory Impact Analysis (RIA). CME = Conventional Maximum Efficiency. CLC = Conventional Lowest Cost. TDB = Total Decarbonization Benchmark. SPD = Slower Partial Decarbonization. FPD = Faster Partial Decarbonization.

**Table 4:**
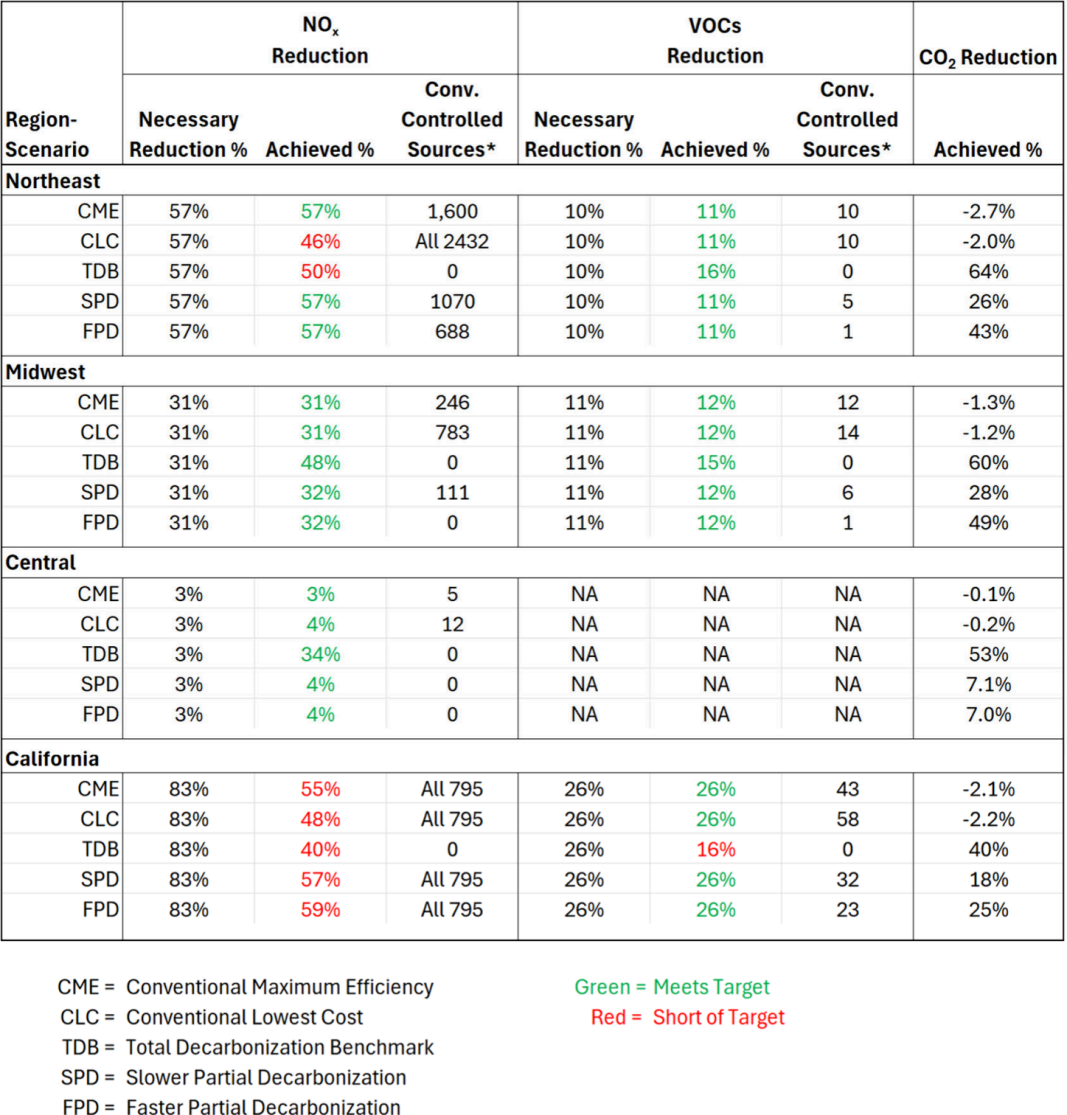
Regional Breakdown of Percent Emissions Changes and Number of Sources Applying Conventional Emission Controls in Each Scenario^a^

a Sources refer to individual facilities or non-point source-type emissions aggregated at county-level. Necessary Reduction % differs slightly from Table 1 because many small sources were truncated from scenario analysis for computational efficiency.

### 3.1 U.S. Regions Are Already Moving Toward Required Emissions Reductions

We find that all five regions have lower NO_*x*_ emissions relative to the RIA Baseline, between 9% and 21% (% Change in Baseline in Table 1, NO_*x*_ section). Four of the five regions have also lowered VOCs emissions, between 0.4 and 15%, except for California where emissions increase 4% (% change in Baseline in Table 1, VOCs section). The Updated Baseline shows all regions have moved closer to the levels needed to meet a 65 ppb ozone standard than projected by EPA a decade ago when the RIA was conducted, with the exception of California VOCs. The Updated Baseline already achieves both the NO_X_ and VOC ETLs in the Southwest and achieves the VOC ETL in the Central Region.

Between 2011 and 2020, the reductions in NO_X_ and VOCs emissions were driven by regulatory controls, the transition to lower-emitting energy resources, technological advancements, and improvements in the transportation sector.^67–69^ Several regulations have targeted NO_X_ and VOCs emissions, including the EPA’s Cross-State Air Pollution Rule and the National Emission Standards for Hazardous Air Pollutants.^70–73^ NO_X_ emissions in the electricity sector have decreased as a result of increased reliance on renewable energy and natural gas in place of coal.^74–76^ NO_X_ and VOCs reductions have also been fueled by developments in control technologies for industry, power plants, and vehicles.^77–80^ The transportation sector has seen a decrease in vehicle emissions due to stricter fuel efficiency standards and tailpipe emission regulations.^81–87^ Additionally, the number of electric and hybrid vehicles has increased, particularly in urban areas.^88–92^

It is worth noting that significant changes in global emissions and climate have occurred since 2011, driven by regional environmental regulations, technological advancements, and shifts in emission sources.^93–95^ While the US and European countries have achieved substantial reductions in anthropogenic NO_X_ and VOC emissions due to emission control policies,^96,97^ Asian countries such as India and regions in the Middle East have experienced increasing NO_X_ emissions due to rapid economic growth and industrialization.^97,98^ NO_X_ emissions in China has been decreased since 2011 following the implementation of stricter regulatory controls,^99^ which led to a decrease in background surface ozone levels in the western U.S. since 2012.^100^ Climate has also warmed over the U.S. since 2011,^101^ although the exact warming since 2011 is difficult to separate from interannual variability associated with the specific meteorology used in the RIA. Ozone has been shown to be the air pollutant most responsive to climate change, with warmer temperatures associated with increased VOC emissions and other processes affecting the efficacy of regulations as well as the contribution of natural and background sources.^102^ These changing conditions, as well as the uncertainty of the original RIA grid modeling, affect the degree to which the calculated emission thresholds reflect real-world conditions needed to achieve a 65 ppb ozone standard.

### 3.2 Conventional Controls Are Effective for NO_*x*_ and VOCs but Increase CO_2_

Figure 2 compares the resulting NO_X_ (upper panel), VOCs (middle panel) and CO_2_ (lower panel) emissions by regions and scenarios. Two scenarios examine conventional control equipment selection choices for meeting the 65 ppb ozone targets: CLC (dark blue bars in Figure 2) and CME (light blue bars in Figure 2). Overall, we find that conventional controls are effective at meeting the ETL, except where the required reduction is especially high for California and Northeast NO_*x*_. We confirm the expected finding that selecting controls for maximum efficiency (CME) meets the ETL, requiring fewer sources to install controls than selecting controls based on the lowest cost (CLC). This result is intuitive, because the lower cost controls have lower emission removal efficiency, thus more sources must be controlled to achieve the same level of reduction. In one instance, for Northeast NO_X_, the maximally controlled CME strategy met the ETL, while the lowest cost CLC strategy could not. Tables 3 and 4 detail the regionally specific observations consistent with these findings.

**Figure 2:**
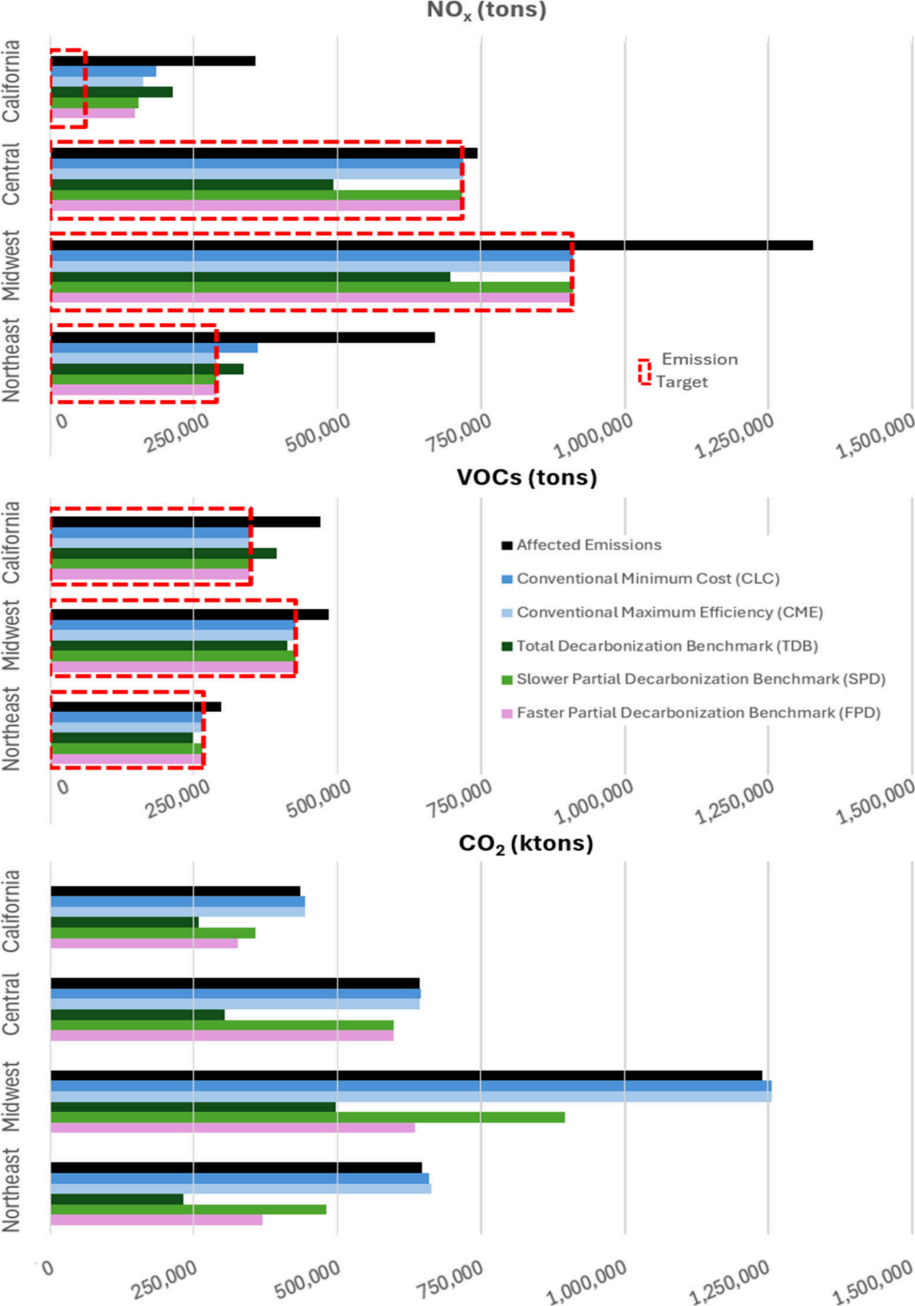
Regional NO_x_, VOCs, and CO_2_ emissions resulting from each scenario. Red dashed rectangles represent inferred emission targets based on analysis of the EPA RIA. Colored bars represent scenario emissions whose magnitude indicates compliance, overcompliance, or under-compliance relative to the respective target.

Where conventional emission controls are applied to fuel combustion sources, CO_2_ emissions increase due to a reduction in thermal efficiency from the auxiliary load of operating the control equipment. We accounted for this increase using heat rate penalties as reported by previous studies. As a result, CO_2_ emissions increase by 1.3% and 1.4% in the CLC and CME scenarios. For the CLC Scenario, the CO_2_ emission increase as a result of thermal efficiency loss is 1.2% for fuel combustion, 1.0% for industrial processes, 2% for mobile sources, and 1.4% for waste disposal sources. For the CME Scenario, the CO_2_ emission increase as a result of thermal efficiency loss is 1.8% for fuel combustion, 1.0% for industrial processes, 1.4% for mobile sources, and 1.2% for waste disposal sources.

While both conventional control scenarios could achieve both NO_X_ and VOCs ETLs in the Northeast and Midwest, they do not address decarbonization priorities relevant to climate change. In fact, conventional controls increase CO_2_ emissions, as well as risking costs from investments into aging infrastructure, i.e., of being stranded assets in the context of energy transition.

### 3.3 Decarbonization Reduces CO_2_ and the Majority of Required NO_x_ and VOCs Reductions, but Requires Some Conventional Controls

We find that the TDB scenario meets required emission targets in the Midwest and Central regions, but not in California (NO_X_ and VOCs) nor in the Northeast (NO_X_).

To consider uncertainty around future decarbonization efforts, the FPD and SPD scenarios limit the sector-specific extent of technology conversion (i.e., fuel switching) to provide a sensitivity comparison. For NO_X_, the FPD Scenario with a higher rate of decarbonization was sufficient to meet Midwest and Central ETLs without any supplemental controls. Meeting the Northeast ETL required supplemental NO_X_ control for 688 sources. In California, no amount of supplemental control could also meet the NO_X_ ETL. For VOCs, the FPD Scenario meets all ETLs, with supplemental controls applied to the largest Midwest and Northeast sources, and the 23 largest California sources. The SPD Scenario, with a slower rate of decarbonization, showed analogous results to its counterpart. Similarly, it was successful in meeting NO_X_ ETLs in all cases except for in California. All other ETLs were similarly achieved, however, in each case the number of supplemental controls increases relative to the FPD Scenario, owing to the lower level of reductions achieved through decarbonization. Comparisons between the number of supplemental controls added for the partial decarbonization scenarios are provided in Table 4. Sources refer to individual facilities or nonpoint source-type emissions aggregated at county-level.

Changes to CO_2_ are shown in Table 2. The level of overall decarbonization is 56% for TDB, 21% for SPD, and 35% for FPD. Regional breakdown of percentage changes is provided in Table 4.

### 3.4 In Decarbonization Scenarios, Emission Reductions Are Relatively Higher for Electricity, Vehicles, And Building Heat, Compared to Using Only Conventional Controls

To compare the impact of decarbonization on the sectoral breakdown of emission reductions, we compare the FPD and the CME scenarios. Figures 3–6 illustrate this comparison for California (Figure 3), Central (Figure 4), Midwest (Figure 5), and Northeast (Figure 6). Major emission sources for NO_X_ and VOCs include fuel combustion for electricity, residential and commercial heating, industrial boilers, noncombustion industrial processes, solvent, light- and heavy-duty vehicles, and other aggregated mobile sources (such as off-road construction equipment, aircraft, marine vessels, and locomotives). As expected, the FPD emission reductions occur in greater proportion for the decarbonized sectors, specifically for electricity, mobile sources (vehicles), and building heat. In the CME scenario, emission reductions occur in greater proportion across all industry, and especially solvent processes for VOCs.

**Figure 3:**
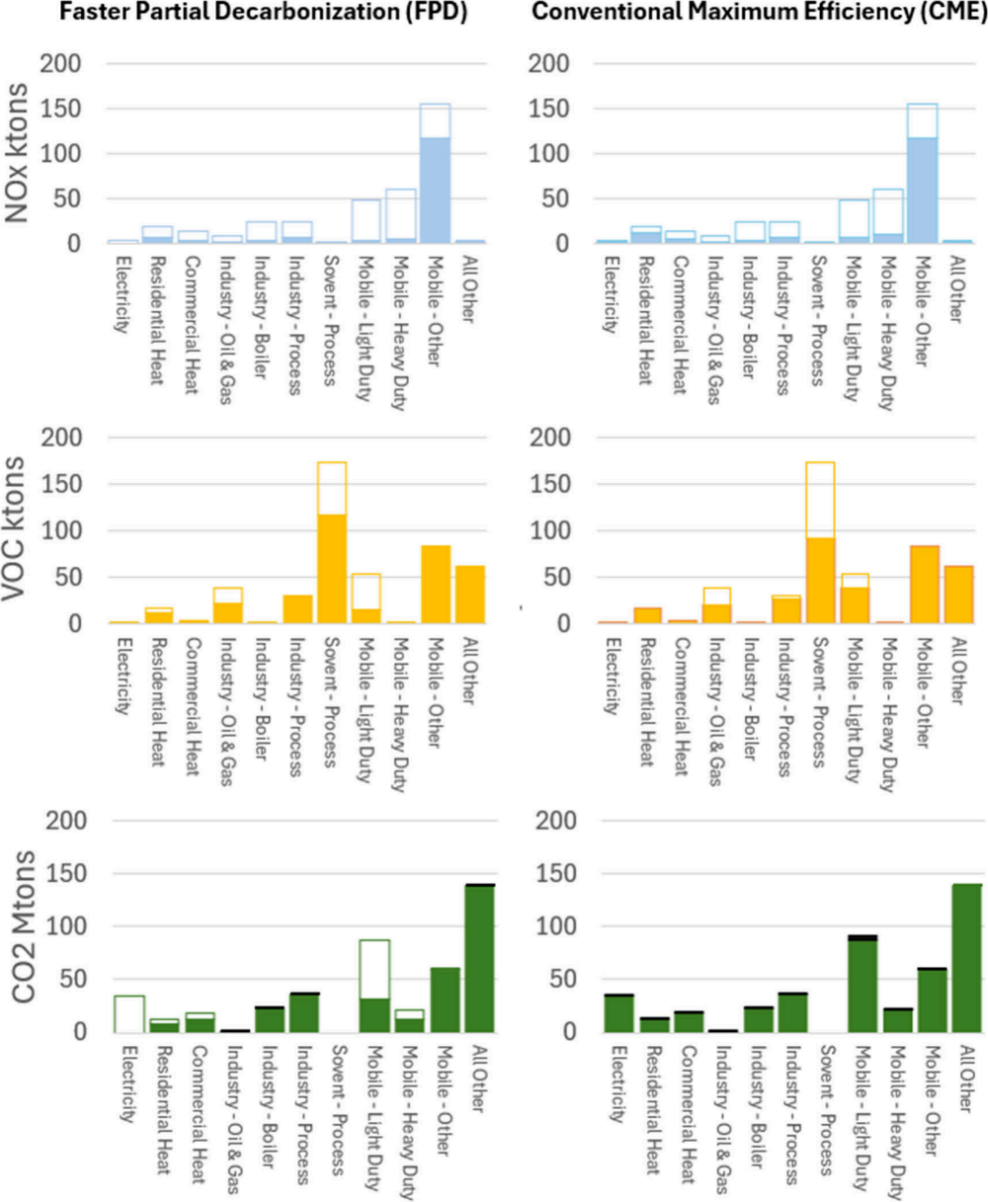
California sectorial emissions comparison for the Faster Partial Decarbonization (FPD) scenario versus the Conventional Maximum Efficiency (CME) Scenario. Unfilled portions of the bars indicate emission reductions baseline, while blackened areas (CO_2_ only) indicate emission increases resulting from the operation of conventional pollution control equipment.

**Figure 4:**
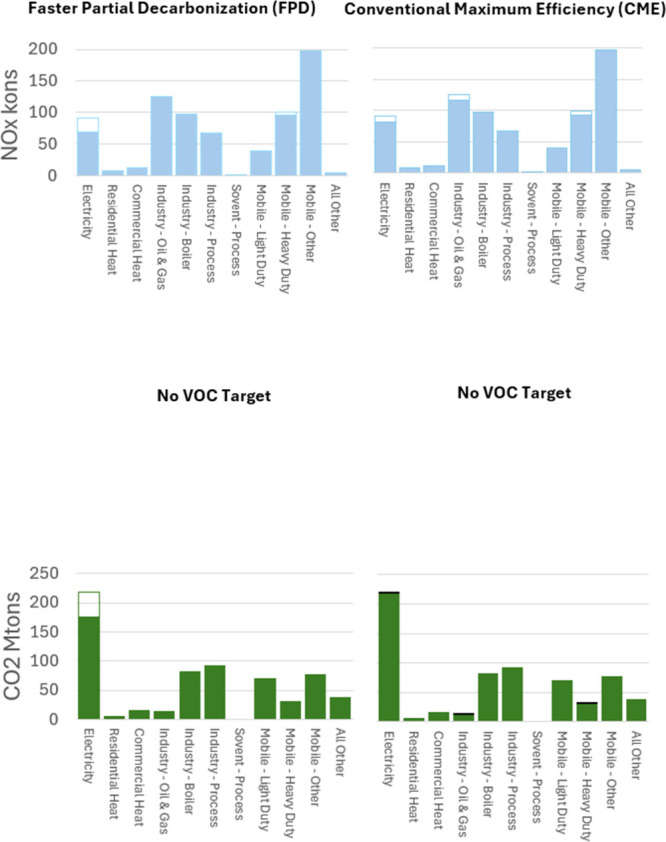
Central sectorial emissions comparison for the Faster Partial Decarbonization (FPD) scenario versus the Conventional Maximum Efficiency (CME) scenario. Unfilled portions of the bars indicate emission reductions baseline, while blackened areas (CO_2_ only) indicate emission increases resulting from the operation of conventional pollution control equipment.

**Figure 5:**
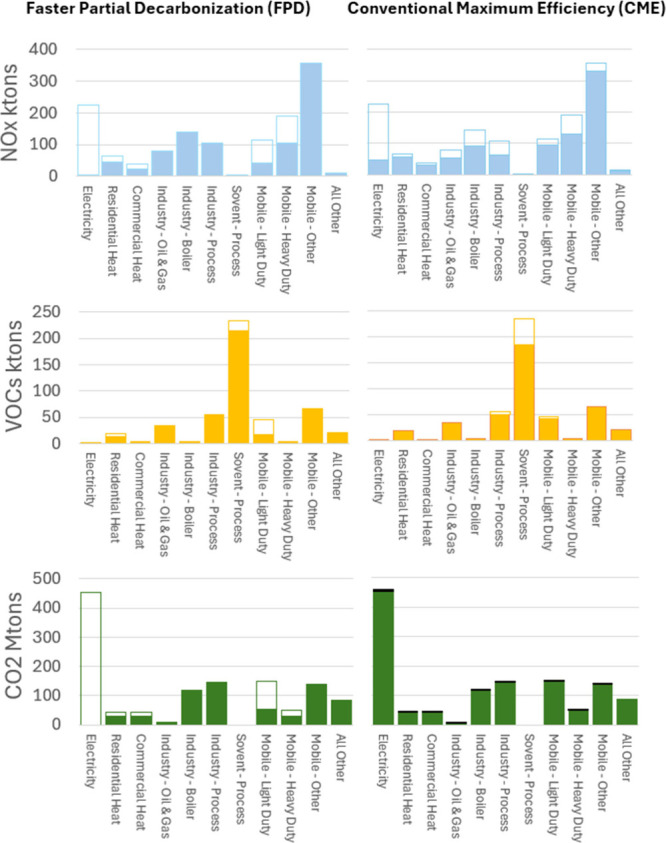
Midwest sectorial emissions comparison for the Faster Partial Decarbonization (FPD) scenario versus the Conventional Maximum Efficiency (CME) scenario. Unfilled portions of the bars indicate emission reductions baseline, while blackened areas (CO_2_ only) indicate emission increases resulting from the operation of conventional pollution control equipment.

**Figure 6:**
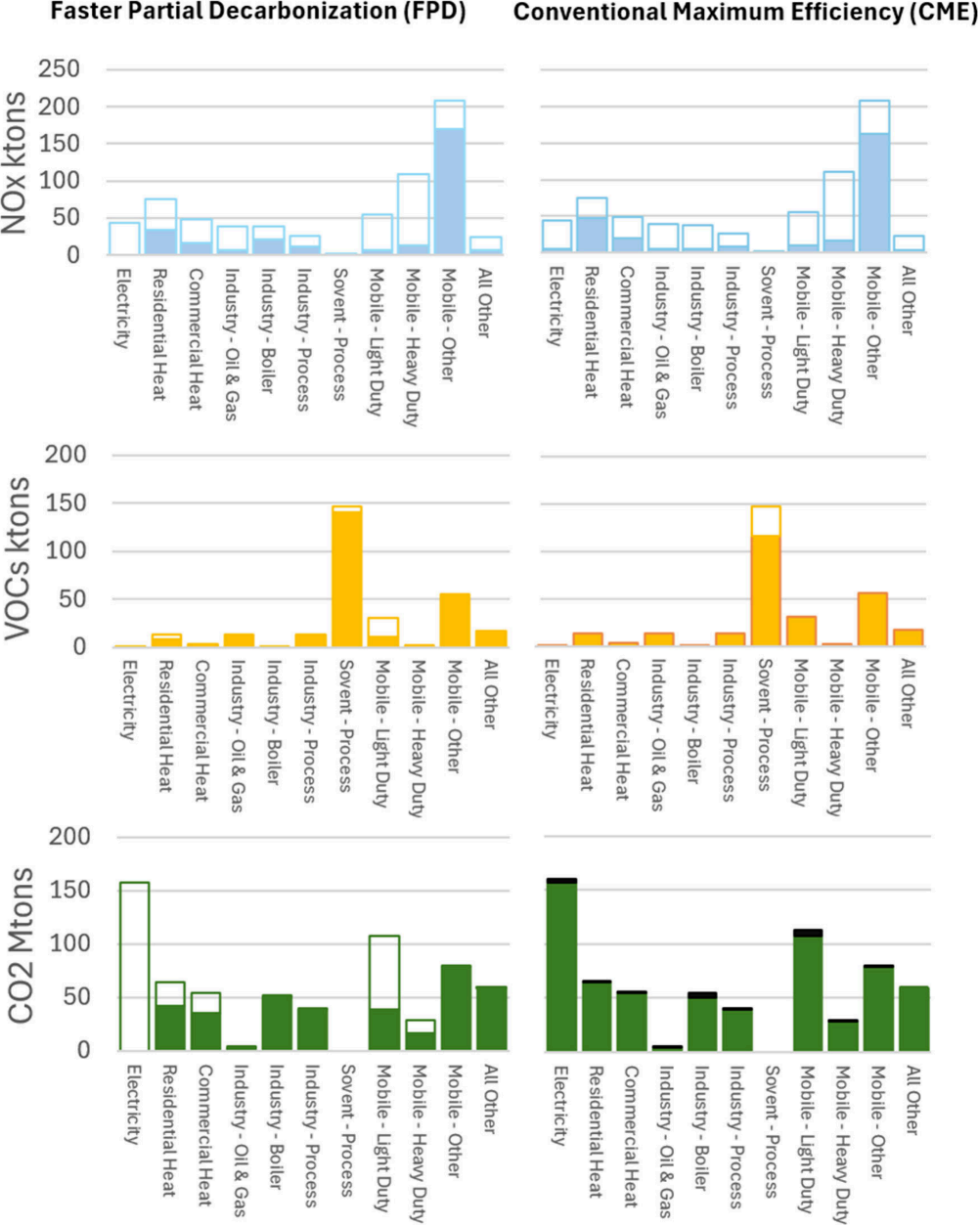
Northeast sectorial emissions comparison for the Faster Partial Decarbonization (FPD) scenario versus the Conventional Maximum Efficiency (CME) scenario. Unfilled portions of the bars indicate emission reductions baseline, while black areas (CO_2_ only) indicate emission increases resulting from the operation of conventional pollution control equipment.

VOCs reductions in the CME scenario occur primarily from solvent processes, with additional contributions from light duty mobile, and oil and gas industry sources. Sources refer to individual facilities or nonpoint source-type emissions aggregated at county-level. For example, county-level “Consumer & Commercial Solvent Use” is the largest single (albeit aggregated) source of VOCs in each of the affected regions. Control options include reduced solvent utilization to avoid VOCs emission via product or process substitution and permanent total enclosures to capture emitted VOCs. Given the importance of this aggregated source and that individual industries may encounter real-world challenges, we recognize that a more nuanced examination of solvent applications and control option feasibility may prove insightful in future work.

Relative to CME, the FPD scenario has a higher proportion of VOCs reductions from light-duty vehicle decarbonization, requiring fewer emission cuts from industrial and solvent processes. In the FPD scenario, a 25% CO_2_ emission reduction occurs across electricity, building heating, and mobile sources, whereas CO_2_ emissions increase 2.1% for the CME scenario. Additional FPD VOCs reductions occur from residential and commercial heat, heavy duty mobile, and electricity sector. Surplus VOCs reduction also occurs in the FPD scenario when fuel substitution reduces both NO_X_ and VOCs in areas that are only required to control NO_X_. While potentially beneficial, these reductions occur outside the areas identified by EPA as most impactful for mitigating ozone formation. Additional details on sectoral changes are provided in Supporting Information Text 2.

## 4 Discussion

The findings of this study highlight the role of decarbonization as a strategy to reduce NO_x_ and VOCs emissions concurrent with CO_2_ reductions. Our main findings suggest that (1) decarbonization alone can achieve the majority of required NO_X_ and VOCs reductions; and (2) conventional controls alone increase emissions of CO_2_.

We quantify regional U.S. emission control scenarios (NO_X_ and VOCs target levels) for a hypothetical 65 ppb ozone standard to examine whether efforts to achieve a tighter ozone standard may be designed to also reduce CO_2_ emissions. We find that the conventional strategies, using technological emission controls, and partial decarbonization pathways, prioritizing decarbonization and supplemented with conventional controls, are both capable of meeting the regional emission targets, except for in California where required NO_X_ reductions exceed what could be achieved either with conventional controls or decarbonization strategies. In particular, the partial decarbonization compliance strategies that prioritize decarbonization, while “filling the gap” with conventional controls, reduce CO_2_ from all affected sources by 21% to 35% depending on slower or faster assumptions for sectoral decarbonization. The slower SPD scenario meets the same number of emission target levels as the faster FPD scenario; however, more sources must implement conventional emission controls in the slower case. Decarbonization is less effective at controlling regional VOCs than NO_X_, in part because technology fuel-switching does not control VOCs from solvent processes, each regions’ largest source of VOCs. Further, because the VOC-controlled regions are geographically smaller than the NO_X_ regions, there is a smaller population of vehicles, buildings, and power plants from which to achieve necessary reductions.

Historically, regulatory air quality management has relied on pollutant-specific control equipment for specific emission sources. Leveraging low-carbon infrastructure to achieve simultaneous emission reductions of multiple pollutants offers an alternative model for air quality management. A drawback to conventional control equipment is that its associated efficiency losses increase CO_2_ emissions. In the conventional scenarios, conventional pollution controls increased CO_2_ emissions by 1.3–1.4% averaged across all regions, and as high as 2.7% for one region in one scenario.

A low-carbon approach avoids the capital and operational costs of emission controls, which can be high. These costs pose longer-term economic risks of stranded assets as expensive controls increase capital investment, and thereby expected lifetime, of fuel combustion sources. Many nonemitting energy technologies, like solar energy and wind energy, are already cost-competitive with conventional fuels,^103,104^ and increased production and innovation are expected to drive prices down further.^105,106^ Ambec and Coria (2018) highlight that “policy spillovers”, through substitution of inputs, shifts in activity levels, or investments in alternative technologies, across pollutants are central to understanding both the efficiency and unintended consequences of regulatory design.

This study demonstrates the technical feasibility for decarbonization to mitigate ozone, using the MECAQC framework to construct pathways to achieve a stricter 65 ppb ozone NAAQS. Building on careful analysis from the 2015 EPA RIA for ozone, and developed expanding on existing EPA regulatory analysis tools, MECAQC provides a comprehensive evaluation of options to achieve emission reduction goals of targeted pollutants (NO_*x*_ and VOCs) with simultaneous evaluation of other pollutants of concern (CO_2_). MECAQC supports analysis of coemitted pollutants, emissions sources, and reduction pathways.

The technical feasibility for each of the scenarios should not be taken lightly. With the exception of TDB, which is intended solely as a benchmark, implementing any scenario has major implications across multiple economic sectors for large areas of the U.S. While CLC focuses on lower cost technologies, it affects many thousands of individual industrial sources, as well as cars, trucks, and energy supply. CME reduces the total number of affected sources, but still has a major sectoral footprint (Refer to Table 4 and Figures 3–6), and at an incrementally higher cost to those sources than CLC. The decarbonization pathways also present feasibility challenges that require the alignment of technological cost effectiveness, political practicality, and societal will. Of these, SPD is more feasible by design than FPD with a reduced rate of sector-specific fuel-shifting. While we focus here on technical feasibility, we recognize the need for detailed cost analysis as part of any policy analysis or air quality management strategy. Prior work with MECAQC for SO_2_ included net costs and benefits of SO_2_ controls;^38^ a comparable approach for NO_*x*_ and VOCs would require more advanced economic analysis. The most significant challenge in the costing of options relates to the comparison of sector-wide decarbonization on regional infrastructure, such as significant expansion of EV charging network or converting buildings to electric heat pumps. The resulting growth in electricity use would require additional investment across generation, transmission, and distribution systems, and would entail significant shifts in usage patterns, such as major increases in winter electricity demand in cold climates.

While air quality improvements are typically thought of as climate “co-benefits”, in fact these cobenefit areas have the potential to be effective drivers of climate action,^107^ across a wide range of environmental and energy areas.^108^ The U.S. Clean Air Act has been driving reductions in emissions since 1970. This powerful legislation offers a pathway to achieve climate goals, by reducing CO_2_ along with regulated emissions of NO_*x*_, VOCs, and other health-relevant pollutants.

## Supplementary Material



## Notes

Detailed scenario results descriptions for decarbonization control strategies, sectoral breakdowns of emissions reductions, a detailed description of the MECAQC framework and underlying data sources, and a baseline emissions table (PDF)
