# Supplementary material for: Emissions Reductions to Meet a Tighter Ozone Standard in the U.S. through Control Technologies versus Clean Energy Transition Scenarios
Source: Environ Sci Technol. 2025 Dec 15;59(50):26999–7012. doi: 10.1021/acs.est.5c01588 (PMC12750530; doi:10.1021/acs.est.5c01588)
Supplement: Supplementary file 1 [file es5c01588_si_001.pdf]

Supplementary Information for

Emissions Reductions to Meet a Tighter Ozone Standard in the U.S. through Control Technologies versus Clean Energy Transition Scenarios

Paul Meier[a*], Tracey Holloway[a,b], Xinran Wu[a], Cecilia Orth[a]

[a] Center for Sustainability and the Global Environment (SAGE), Nelson Institute for Environmental Studies, University of Wisconsin-Madison, 1710 University Avenue, Madison, WI 53726, United States

[b] Department of Atmospheric and Oceanic Sciences, University of Wisconsin-Madison, 1225 West Dayton Street, Madison, WI 53706, United States

Corresponding author email: pmeier@wisc.edu

Contents
Pages = 7, Text = 2, Table = 1, Figure = 1

Supplementary Text S1. Detailed Scenario Explanations of Decarbonization Scenario Results

We evaluated three scenarios that relied exclusively on decarbonization and found that decarbonization can achieve most required NO_x and VOCs reductions. The Total Decarbonization Benchmark (TDB) scenario assumes the removal of all fuel combustion emissions from electricity, vehicles and building heating within targeted regions. We recognize that complete decarbonization over the next 15 years may be viewed an unrealistic policy objective, to varying degrees, across different sectors. Therefore, we constructed two partial decarbonization scenarios to evaluate sensitivity around the extent of technology conversion (i.e., fuel switching), applied within specific sectors. The Faster Partial Decarbonization (FPD) assumes that 100% decarbonization for the electricity sector is achievable, as in the TDB scenario. More conservatively, however, FPD assumes an upper limit of 45% decarbonization for heavy-duty vehicles, 65% for light-duty vehicles, and 36% building electrification. For the Slower Partial Decarbonization Scenario (SPD), decarbonization is limited to half the fast sectoral rate: 50% decarbonization for electricity, 22.5% for heavy-duty vehicles, 32.5% for light-duty vehicles, and 18% building electrification. We refer to the partial decarbonization scenarios collectively as “blended” compliance strategies, because both use decarbonization along with the minimum use of conventional controls required across all regions and standards. For blended scenarios, decarbonization is applied only up to the point where ETLs are met.

Where ETLs are not met, supplemental emission controls are added up to the point to meeting the target, or supplemental control options are exhausted (only in the case of California NO_x).

For NO_x emissions (**Figure 2**, upper), the TDB scenario (dark green bars) could achieve the NO_x ETL in Midwest, and Central, but not the Northeast or California. By supplementing with conventional controls the FPD and SPD scenario (light green and pink bars) achieves the NO_x ETL in Midwest and Central, as well as in the Northeast, but still not in California. Similarly, Figure 2 middle panel compares VOCs emissions. All decarbonization scenarios achieve the VOCs ETL in Midwest and Northeast regions, but only the partial decarbonization scenarios meet California's target, with the help of conventional controls. The Central region does not have a VOCs ETL because it was already met in the Updated Baseline.

Decarbonization strategies achieve greater NO_x reductions than VOCs reductions, because of the co-emission of NO_x and CO_2 through combustion processes. Figure 2 lower panel compares CO_2 emissions. Across all affected regions, total decarbonization, faster partial, and slower partial decarbonization results in a 56%, 35%, and 21% reduction in CO_2 emissions, respectively, by eliminating combustion emissions. TDB reduces 1.67 billion tons, FPD reduces 1.03 billion tons, and SPD reduces 0.63 billion tons of $CO_{2,}$ respectively.

California - The TDB scenario reduces California's affected NO_x emission by 40% and VOCs by 16%; whereas the SPD and FPD scenarios reduces California's NO_x by 57% and 59%, respectively, and VOCs by 26%. The California region does not achieve NO_x or VOCs ETLs under total or partial decarbonization. The challenge of meeting California's NO_x target is the depth of the desired reductions, requiring an 82% reduction. Total decarbonization achieves less than two-thirds of necessary VOCs reductions, while partial (blended) decarbonization scenarios achieve the VOC target with the aid of supplemental conventional controls. The TDB scenario reduces California's CO_2 emission by 40% while the SPD and FPD scenario reduces CO_2 by 18% and 25%, respectively. Decarbonization is less productive in California than other regions because California has a much lower proportion of CO_2 emissions occurring from the electricity sector.

Central - The TDB scenario reduces the Central region's affected NO_x emission by 34%, compared to 4% in the FPD and SPD scenarios. Partial decarbonization scenarios limit the total sectoral fuel-switch so as to not exceed the Central region's modest NO_x reduction requirement (3.5%). There is no VOCs ETL for the Central region, having met the desired levels in the Updated Baseline. The Central region's affected CO_2 emissions are reduced by 53% in the TDB scenario and 7% in FDP and SPD scenarios.

Midwest - The TDB scenario reduces the Midwest's affected NO_x emissions by 48% and VOCs by 15%, whereas the SPD and FPS scenarios reduces NO_x and VOCs by 32% and 12%, respectively. The Midwest's affected CO_2 emissions are reduced by 60% in the TDB scenario, 28% in SPD and 49% in FPD.

Northeast - The TDB scenario reduces the Northeast's affected NO_x emission by 50% and VOCs by 16%, whereas the SPD and FPD reduces NO_x by 57% and VOCs by 11%. TDB meets the Northeast VOCs ETL, but not the NO_x ETL, achieving only 88% of necessary NO_x reductions. This is because Northeast has a challenging NO_x ETL (57% necessary reduction). Both blended partial decarbonization scenarios achieve the necessary 11% NO_x reductions and the necessary 10% VOCs reductions. The Northeast's affected CO_2 emissions are reduced by 64% in the TDB scenario, by 26% in SPD, and 43% in PDB.

Sectoral Examination of Reductions

Sectorial breakdown of emission reductions is shown in **Figure 3** comparing the FPD and CME scenarios, for California (3A), Central (3B), Midwest (3C), and Northeast (3D). Major emission sources for NO_x and VOCs include fuel combustion for electricity, residential and commercial heating, industrial boilers, non-combustion industrial processes, solvent, light- and heavy-duty vehicles, and other aggregated mobile sources (such as off-road construction equipment, aircrafts, marine vessels, and locomotives). Unsurprisingly, the FPD emission reductions occur in greater proportion for the decarbonized sectors, specifically for electricity, mobile sources (vehicles), and building heat. In the CME scenario, emission reductions occur in greater proportion across all industry, and especially solvent processes for VOCs.

VOCs reductions in the CME scenario occur primarily from solvent processes, with additional contributions from light-duty mobile, and oil and gas industry sources. Relative to CME, the FPD scenario has a higher proportion of VOCs reductions from light-duty vehicle decarbonization, requiring fewer emission cuts from industrial and solvent processes. In the FPD scenario, a 25% CO_2 emission reduction occurs across electricity, building heating, and mobile sources, whereas CO_2 emissions increase 2.1% for the CME scenario. Additional FPD VOCs reductions occur from residential and commercial heat, heavy duty mobile, and electricity sector. Surplus VOCs reduction also occurs in FPD scenarios when fuel substitution reduces both NO_x and VOCs in areas that are only required to control NO_x. While potentially beneficial, these reductions occur outside the areas identified by EPA as most impactful for mitigating ozone formation.

California - Most of California's NO_x reductions occur from mobile sources, including heavy-duty, light-duty vehicles, and some non-road equipment in the FPD (66% of NO_x reduction) and CME (67% of NO_x reduction). However, neither scenario achieves the California NO_x ETL by controlling all applicable sources, which would require an 83% reduction in affected emissions.

Central - The Central region's sectoral breakdown is shown in **Figure 3B**. In the FPD, most of the NO_x reductions (85%) occur from decarbonization of the electricity sector. In contrast, NO_x reductions in the CME are evenly distributed between the oil and gas industry (36%), electricity sector (34%), and mobile sources (30%). The Central region has no VOCs ETL.

Midwest - Sectorial breakdown of Midwest emission reductions is shown in **Figure 3C**. In the Blended compliance strategy, Midwest VOCs reductions are derived from mobile sources (52%) and solvent processes (33%, including supplemental controls) with lesser contributions from building heat and electricity. In contrast, the CME scenario achieves nearly all (93%) of its reductions through control of solvent processes and other industrial sources.

Northeast - Because of the Northeast's steep 55% necessary NO_x reduction, the FPD and CME deploy nearly all control options considered, resulting in highly similar sectoral profiles in Figure 3D. The same is not true for VOCs where the CME scenario relies exclusively (100%) on solvent processes, while the FPD implements VOCs reductions more widely across mobiles sources (63%), solvent processes (19% including supplemental controls), building heat (16%), and electricity sector (2%).

Supplementary Text S2. Detailed Description of MECAQC

MECAQC is a spreadsheet-based framework that integrates information on control technologies, control efficiencies, and average abatement costs from established sources. Conventional control technologies, including end-of-pipe devices (e.g., oxidizers for VOCs) and process modifications (e.g., adjusting air-to-fuel ratios to reduce vehicular NOx emissions), are derived from the EPA Air Pollution Control Cost Manual,[1] the CoST dataset,[2] and peer-reviewed literature.[3] Energy transition technologies, such as fuel switching from coal to natural gas or renewables, deployment of electric vehicles, and efficiency improvements, are also represented with associated reductions in pollutant emissions and CO_2. These datasets and relevant information from the literature are combined to come up with sector-specific pollution reduction pathways, to form the foundation of our scenarios and to evaluate the availability and efficiency of pollution control for VOCs and NOx controls across most source categories.

The EPA Air Pollution Control Cost Manual includes cost estimations and engineering configurations (*e.g.* gas flow rate and control efficiency) of particular point source and stationary area source pollution controls for NO_x and VOCs. The CoST data includes control efficiencies, cost-per-ton estimates, and associated source sectors of different air pollution controls at facility level. From an electricity generation perspective, pollution controls act as an auxiliary load, reducing overall system efficiency and increasing fuel combustion. To account for the increasing CO_2 emissions caused by the efficiency loss from operating pollution controls, we use an average value from 0.5% to 0.7% based on previous studies.[4,5] The energy transition technologies considered include a series decarbonization actions such as fuel-switching from coal to natural gas,[6,7] and from coal to renewable energy,[8–11] electric vehicles (EVs),[12–14] and energy efficiency improvement.[15] These technologies could reduce both air pollutants and CO_2 emissions by about 15%-100%, depending on the scale of implementation.[14,15] Life-cycle impacts are not included in this study, as

it focuses solely on direct emissions at the point of combustion. Fuel-cycle assumptions for natural gas fuel-switching can significantly influence net GHG emissions, primarily due to methane leakage.[16] However, such considerations fall outside the scope of this study.

Supplemental Table S1. **Fused Baseline emissions combining NEI and other appropriate emissions data.**

Merged Baseline Emissions U.S. 48 States	NOx (Ktons)	VOC (Ktons)	GHG* (Ktons)	Baseline Data Source
Mobile	3,721	1,996	1,905,497	Onroad Vehicles from from EPA MOVES (2023). Nonroad vehicle NOx and VOC from EPA NEI (2017) and Nonroad CO2 from EIA(2020).
Fuel Combustion	2,134	461	2,988,549	Electricity sector NOx and CO2 from EPA CAMD (2021). Electricity VOC from EPA NEI scaled based on NOx. Non-electricity fuel combustion NOx and VOC from EPA NEI (2017) and Non-electricity CO2 from EIA(2020).
Industrial Processes	1,167	3,121	734,937	NOx and VOC from EPA NEI (2017) and GHG from EPA GHG (2020).
Miscellaneous Non-Industrial	3	99	8,733	NOx and VOC from EPA NEI (2017) and GHG from EPA GHG (2020).
Solvent	3	2,935	0	NOx and VOC from EPA NEI (2017) and GHG from EPA GHG (2020).
Pet Terminals & Gas Stations	0	564	45	NOx and VOC from EPA NEI (2017) and GHG from EPA GHG (2020).
Agriculture	0	228	671,069	NOx and VOC from EPA NEI (2017) and GHG from EPA GHG (2020).
Waste Disposal	81	171	161,145	NOx and VOC from EPA NEI (2017) and GHG from EPA GHG (2020).
**Grand Total**	**7,109**	**9,576**	**6,469,974**	

* On-road vehicles and fuel combustion are reported as CO_2. All other emissions are reported as CO_2-equivalent.

References:

(1) U.S. EPA. *EPA Air Pollution Control Cost Manual*. https://www.epa.gov/economic-and-cost-analysis-air-pollution-regulations/cost-reports-and-guidance-air-pollution#cost%20manual (accessed 2024-01-16).

(2) Community Modeling and Analysis System. *Control Strategy Tool (CoST)*. https://www.cmascenter.org/cost/ (accessed 2024-03-19).

(3) Wang, L.; Patel, P. L.; Yu, S.; Liu, B.; McLeod, J.; Clarke, L. E.; Chen, W. Win–Win Strategies to Promote Air Pollutant Control Policies and Non-Fossil Energy Target Regulation in China. *Applied Energy 163*, 244–253. https://doi.org/10.1016/j.apenergy.2015.10.189.

(4) Brown, M. A.; Li, Y.; Massetti, E.; Lapsa, M. U.S. Sulfur Dioxide Emission Reductions: Shifting Factors and a Carbon Dioxide Penalty. *The Electricity Journal* **2017**, *30*, 17–24. https://doi.org/10.1016/j.tej.2016.12.007.

(5) Schuster, E.; Romero, C.; Yao, Z.; Si, F. Integrated Real-Time Optimization of Boiler and Post-Combustion System in Coal-Based Power Plants via Extremum Seeking. In *Proceedings of the IEEE International Conference on Control Applications*; 2010; pp 2184–2189. https://doi.org/10.1109/CCA.2010.5611176.

(6) Lueken, R.; Klima, K.; Griffin, W. M.; Apt, J. The Climate and Health Effects of a USA Switch from Coal to Gas Electricity Generation. *Energy* **2016**, *109*, 1160–1166. https://doi.org/10.1016/j.energy.2016.03.078.

(7) de Gouw, J. A.; Parrish, D. D.; Frost, G. J.; Trainer, M. Reduced Emissions of CO2,NOx,and SO2 from U.S. Power Plants Owing to Switch from Coal to Natural Gas with Combined Cycle Technology. *Earth'sFuture* **2014**, *2*, 75–82. https://doi.org/10.1002/2013ef000196.

(8) Abel, D.; Holloway, T.; Harkey, M.; Rrushaj, A.; Brinkman, G.; Duran, P.; Janssen, M.; Denholm, P. Potential Air Quality Benefits from Increased Solar Photovoltaic Electricity Generation in the Eastern United States. *Atmospheric Environment* **2018**, *175*, 65–74. https://doi.org/10.1016/j.atmosenv.2017.11.049.

(9) Alvarez-Herranz, A.; Balsalobre-Lorente, D.; Shahbaz, M.; Cantos, J. M. Energy Innovation and Renewable Energy Consumption in the Correction of Air Pollution Levels. *Energy Policy* **2017**, *105*, 386–397. https://doi.org/10.1016/j.enpol.2017.03.009.

(10) Peng, W.; Yang, J.; Wagner, F.; Mauzerall, D. L. Substantial Air Quality and Climate Co-Benefits Achievable Now with Sectoral Mitigation Strategies in China. *Science of the Total Environment* **2017**, *598*, 1076–1084. https://doi.org/10.1016/j.scitotenv.2017.03.287.

(11) Zhu, Y.; Wang, Z.; Yang, J.; Zhu, L. Does Renewable Energy Technological Innovation Control China's Air Pollution? A Spatial Analysis. *Journal of Cleaner Production* **2020**, *250*, 119515. https://doi.org/10.1016/j.jclepro.2019.119515.

(12) Alimujiang, A.; Jiang, P. Synergy and Co-Benefits of Reducing CO2 and Air Pollutant Emissions by Promoting Electric Vehicles—A Case of Shanghai. *Energy for Sustainable Development* **2020**, *55* (181–189). https://doi.org/10.1016/j.esd.2020.02.005.

(13) Duan, S.; Qiu, Z.; Liu, Z.; Liu, L. Impact Assessment of Vehicle Electrification Pathways on Emissions of CO2 and Air Pollution in Xi'an, China. *Science of the Total Environment* **2023**, *893*, 164856. https://doi.org/10.1016/j.scitotenv.2023.164856.

(14) Peters, D. R.; Schnell, J. L.; Kinney, P. L.; Naik, V.; Horton, D. E. Public Health and Climate Benefits and Trade-Offs of U.S. Vehicle Electrification. *GeoHealth* **2020**, *4*. https://doi.org/10.1029/2020GH000275.

(15) Abel, D.; Holloway, T.; Martínez-Santos, J.; Harkey, M.; Tao, M.; Kubes, C.; Hayes, S. Air Quality-Related Health Benefits of Energy Efficiency in the United States. *Environmental Science & Technology* **2019**, *53*, 3987–3998. https://doi.org/10.1021/acs.est.8b06417.

(16) Gordon, D.; Reuland, F.; Jacob, D. J.; Worden, J. R.; Shindell, D.; Dyson, M. Evaluating Net Life-Cycle Greenhouse Gas Emissions Intensities from Gas and Coal at Varying Methane Leakage Rates. *Environ. Res. Lett.* **2023**, *18* (8), 084008. https://doi.org/10.1088/1748-9326/ace3db.
